# Accuracy of intraoperative identification of the tibial intercondylar eminence in the sagittal plane in dogs

**DOI:** 10.1111/vsu.14285

**Published:** 2025-06-02

**Authors:** Brenda M. Alcântara, Bruno W. Minto, Alefe L. C. Carrera, Rodrigo C. S. Faustino, Lara C. M. Lopes, Luis G. G. G. Dias

**Affiliations:** ^1^ Department of Veterinary Clinic and Surgery School of Agricultural and Veterinarian Sciences, São Paulo State University Jaboticabal Brazil

## Abstract

**Objective:**

To assess the accuracy of intraoperative identification of the tibial intercondylar eminence (TIcE) in the sagittal plane in dogs.

**Study design:**

Ex vivo experimental study.

**Animals:**

A total of 20 stifles from 10 dogs (weight range: 20–30 kg; *n* = 20).

**Methods:**

Stifles were positioned for a mediolateral radiographic projection with a hypodermic needle placed at the center of the medial collateral ligament (MCL) (C), immediately cranial (Cr), and caudal (Cd) to the ligament. Variables were assessed at the stifle flexions of 90° and 135° with both intact cranial cruciate ligament (CCL‐In) and after mechanical transection (CCL‐MT). Three evaluators measured the distance (*d*) between the TIcE and needle center. Statistical analysis involved a linear mixed model, with the Bonferroni test (*p* < .0125).

**Results:**

Analyses of CCL‐In and CCL‐MT groups revealed statistically significant differences between needle positions and stifle flexion angles. In the CCL‐In group, the C‐90° position was closest to the stifle center (*d* = 0.45 ± 2.39). For the CCL‐MT group, the C‐135° position was nearest (*d* = 0.11 ± 2.18).

**Conclusion:**

The center of the MCL in the sagittal plane, at 135° of stifle flexion, served as a reliable anatomical reference for identifying the TIcE in dogs with CCL disease.

**Clinical significance:**

A meticulous intraoperative identification of the TIcE can improve the accuracy of tibial osteotomies, potentially optimizing tibial plateau leveling osteotomy outcomes.

## 1 INTRODUCTION

Tibial plateau leveling osteotomy (TPLO) is a widely used surgical technique for treating cranial cruciate ligament (CCL) insufficiency in dogs, targeting dynamic stabilization of the stifle and neutralizing cranial tibial thrust.^1^, ^2^, ^3^, ^4^ Preoperative TPLO planning is critical, thorough, and standardized, yet its success relies on accurate intraoperative transposition of measurements.^5^ The surgeon's skill in replicating these measurements directly influences procedural success, optimizing clinical outcomes.^6^, ^7^

Radial osteotomy is a pivotal step in TPLO, as it affects the postoperative tibial plateau angle (TPA) and the geometric configuration of the remaining tibial tuberosity. Misalignment can lead to complications such as tibial tuberosity fractures.^6^, ^8^, ^9^ Osteotomy centered on the tibial intercondylar eminence (TIcE) is the most precise anatomical location, facilitating TPLO's geometric, biomechanical, and surgical objectives. Therefore, reliable intraoperative identification of the TIcE in the sagittal plane is essential.^10^

Standard anatomical landmarks, including the tibial tuberosity, patellar ligament insertion, and the proximal tibial caudal edge, are frequently used for surgical replication.^11^, ^12^ The medial collateral ligament (MCL) of the stifle provides a crucial reference for TIcE identification, particularly at the proximal tibia's medial edge.^13^, ^14^, ^15^, ^16^ However, while some studies have demonstrated the MCL insertion site and tension variations according to stifle positioning in dogs, no studies have objectively evaluated the precise anatomical and radiographic relationship between the MCL and the TIcE, which may be critical for accurate TPLO technique execution.^17^, ^18^, ^19^

This study aimed to identify the most accurate anatomical landmark on the MCL for TIcE identification, representing the stifle joint center (defined as the rotation center in TPLO planning). Additionally, we investigated the effects of stifle flexion/extension and CCL integrity. We hypothesized that the MCL center in the sagittal plane corresponds anatomically to the TIcE and that stifle flexion amplitude and CCL insufficiency influence this correspondence.

## 2 MATERIALS AND METHODS

### 2.1 Animals

This study used 10 canine cadavers weighing 20–30 kg. Death causes were unrelated to the study, and owner permission was obtained. Both stifles from each cadaver were analyzed, yielding 20 stifles (*n* = 20). The study protocol was approved by the Ethics Committee for Animal Use at FCAV UNESP (protocol no. 2264/2024). Exclusion criteria included previous stifle surgery, CCL insufficiency, bone lesions in the distal femur or proximal tibia, or severe osteoarthrosis that prevented proper anatomical and radiographic landmark identification.

With each dog in lateral recumbency and the pelvic limb parallel to the table, the stifle and proximal tibia were accessed via a medial incision from the patella to the end of the tibial tuberosity. After elevating the pes anserinus, the MCL was identified, and its width was measured at the joint level using a Castroviejo caliper. Medial parapatellar arthrotomy was then performed, and the CCL was mechanically transected using a no. 24 scalpel blade.

### 2.2 Radiographs

Radiographs were obtained using a portable X‐ray unit (Poskom 20BT) and a direct revealer (DR) plate device (DR Mars 1417X) with settings at 1 mAs and 60 kV. Each stifle was positioned in lateral recumbency with the tibia in a mediolateral orientation parallel to the DR plate for TPA measurement and TIcE identification. A 1‐inch radiographic magnifier, positioned at the same height as the proximal tibia, ensured image accuracy. A goniometer was used to ensure precise stifle flexion angles (90° or 135°).

**FIGURE 1:**
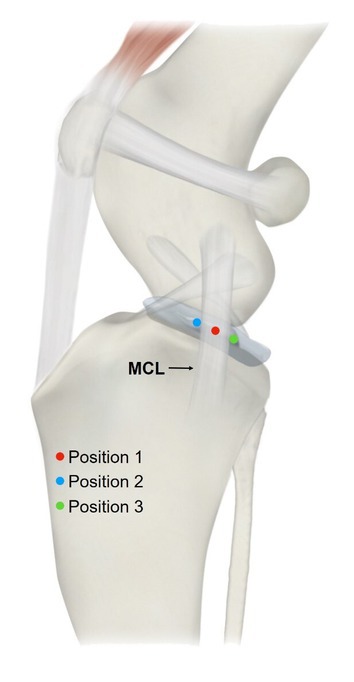
Schematic illustration of the medial view of a canine stifle, showing the medial collateral ligament (MCL) as a reference for needle insertion into the stifle joint (position 1 – center [C], position 2 – cranial [Cr], or position 3 – caudal [Cd]).

In the mediolateral projection, initial imaging was conducted at 90° stifle flexion to measure TPA, followed by group‐specific imaging to evaluate needle positioning relative to the MCL. The needle was inserted perpendicular to the sagittal plane and positioned proximal to the tibial plateau at three locations: MCL center (position 1 – C), cranial to the MCL (position 2 – Cr), and caudal to the MCL (position 3 – Cd) (Figure 1). Imaging was first conducted at 90° flexion and then repeated at 135° flexion, both for intact CCL (CCL‐In) and mechanically transected CCL (CCL‐MT) conditions. The needle was consistently inserted parallel to the X‐ray beam, producing a point‐like image.

**FIGURE 2:**
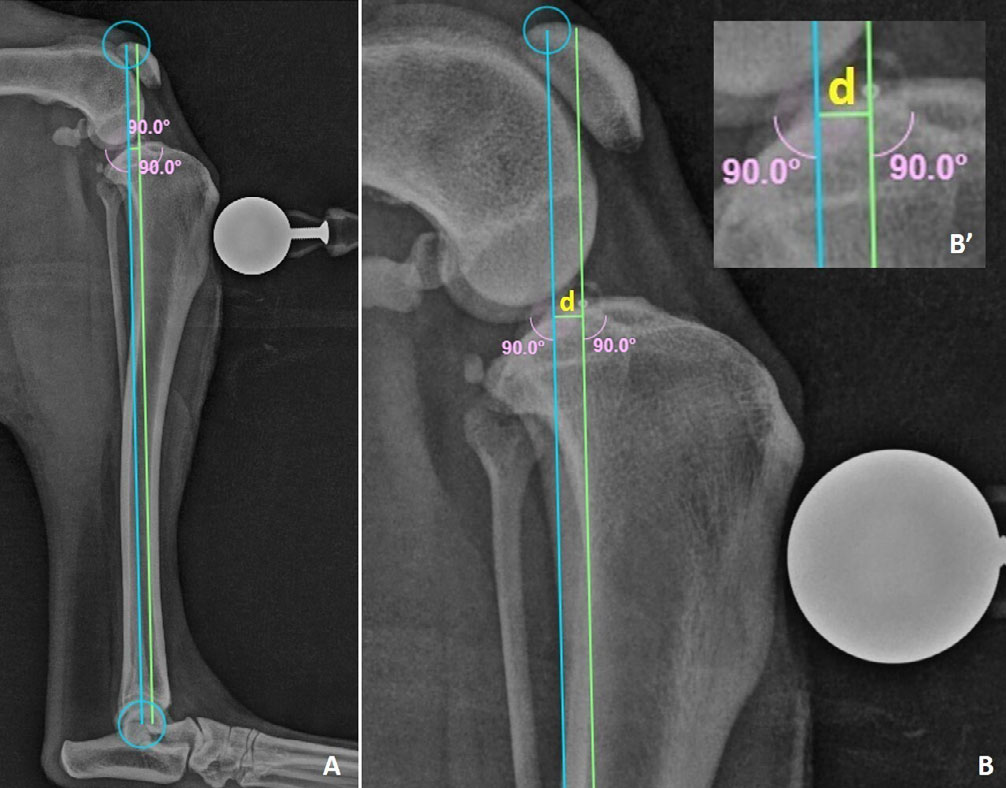
Radiographic images of a canine tibia in mediolateral projection with 90° stifle flexion. (A) Mechanical tibial axis (blue line) with a parallel line (green line) positioned at the needle center. (B) Perpendicular distance (“*d*”) illustrating the distance from the needle to the tibial intercondylar eminence (B′).

Radiographic images were evaluated by three expert orthopedic surgeons using vPOP Pro software (VETSOS Education Ltd., Shrewsbury, UK). A standardized measurement protocol was used in which the tibial mechanical axis was determined, and a parallel line was drawn through the needle center. The perpendicular distance between these two lines was termed “*d*” and represented the distance from the needle position to the TIcE (Figure 2). When the lines aligned, indicating the needle was at the TIcE, “*d*” was recorded as 0. If the needle was cranial to the TIcE, a negative value was recorded; if caudal, a positive value was recorded. Each evaluator analyzed 240 radiographs from 20 canine stifles across the needle positions (C, Cr, and Cd) at two flexion angles (90° and 135°) and two CCL conditions (CCL‐In and CCL‐MT).

### 2.3 Statistical analysis

Agreement between evaluators on distance classification across 240 radiographs was assessed using the intraclass correlation coefficient (ICC). The ICC analysis aimed to generalize the agreement results to evaluators with qualifications comparable to those of the three evaluators in this study, enhancing its applicability in clinical trials. It was calculated via a two‐way random‐effects model, where both cases and evaluators were considered random, to determine absolute agreement. ICC interpretation was based on established thresholds: a score of 0 indicated no agreement, and a score of 1 indicated perfect agreement. ICC values were classified as follows: >0.9 indicated excellent agreement, 0.75–0.89 indicated good agreement, 0.50–0.74 indicated moderate agreement, and <0.50 indicated poor agreement.^20^

Descriptive statistics were calculated for the variable “*d”* to obtain the mean, SD, and SE values. A linear mixed‐effects model was applied to assess the effects of needle position, stifle flexion angle, and CCL characteristics on “*d”*, with each dog treated as a random effect. The model included needle position and stifle flexion angle as fixed effects and their interaction. Model assumptions were evaluated by examining residuals using the Shapiro–Wilk and Levene's tests, followed by normal quantile plots for normality and scatter plots of residuals versus fitted values for homoscedasticity. Multiple comparisons among needle positions (C, Cr, and Cd) and stifle flexion angles (90° and 135°) were performed using estimated marginal means, with significance adjusted via the Bonferroni correction. All analyses were conducted using R software,^21^ with a significance level of 5%.

## 3 RESULTS

The study included 10 canine cadavers, comprising 20 stifles. A total of 60% of the cadavers were male, and 50% were mixed breed. The mean bodyweight was 23.17 ± 3.21 kg. MCL widths were 5.60 ± 0.84 mm for the right stifle and 5.55 ± 1.01 mm for the left stifle. Mean TPAs for evaluators 1–3 were 24.5° ± 3.8°, 25.8° ± 3.9°, and 25.1° ± 2.5°, respectively.

**FIGURE 3:**
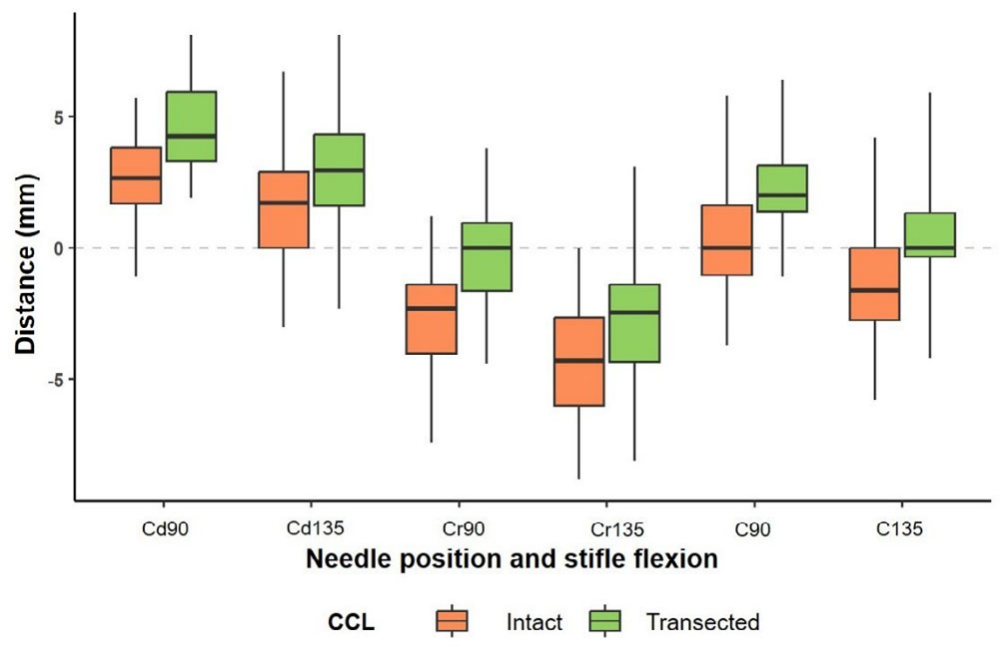
Box plot showing the combined statistical analysis of the intact cranial cruciate ligament (CCL‐In) and mechanically transected cranial cruciate ligament (CCL‐MT) groups by needle position and stifle flexion angle, illustrating the distance “*d*” from the needle to the stifle center.

A *p*‐value < .0001 indicated statistically significant interevaluator agreement. The ICC value of 0.92 (95% CI: 0.83–0.95) demonstrated high agreement across evaluators. CCL‐In and CCL‐MT groups were analyzed independently, and in both groups, there was a significant effect in the interaction between needle position and the stifle flexion angle. A significant difference (*p* < .0125) was observed between mean needle position and stifle flexion angle across all subgroups (Table 1). For the CCL‐In group, the needle position closest to the stifle joint center and TIcE was C‐90° (mean *d* = 0.45 ± 2.39), while for the CCL‐MT group, it was C‐135° (mean *d* = 0.11 ± 2.18). A distinct pattern emerged, with mean “*d*” values for stifle flexion in the CCL‐In group being significantly lower than those in the CCL‐MT group (*p* < .0125; Figure 3).

**TABLE 1:** Summary statistics for distance “*d*” (mm) based on CCL characteristics, needle position, and stifle flexion angle.

CCL	Needle position	Stifle flexion angle	Radiographs (*n*)	Mean ± SD	SE	Min	Max
Intact	Cd	90	60	2.73 ± 1.79^f^	0.37	−1.1	5.7
		135	60	1.52 ± 2.18^e^	0.37	−3	6.7
	Cr	90	60	−2.58 ± 1.88^b^	0.37	−7.4	1.2
		135	60	−4.27 ± 2.08^a^	0.37	−8.8	0
	C	90	60	0.45 ± 2.39^d^	0.37	−3.7	5.8
		135	60	−1.12 ± 2.46^c^	0.37	−5.8	4.2
Mechanically transected	Cd	90	60	4.58 ± 1.71^f^	0.37	1.9	8.1
		135	60	2.86 ± 2.13^e^	0.37	−2.3	8.1
	Cr	90	60	−0.18 ± 1.81^b^	0.37	−4.4	3.8
		135	60	−2.80 ± 2.39^a^	0.37	−8.1	3.1
	C	90	60	2.33 ± 1.75^d^	0.37	−1.1	6.4
		135	60	0.11 ± 2.18^c^	0.37	−4.2	5.9

*Note*: Lowercase letters denote statistically significant difference (*p* < .0125) in the mean values for needle position and stifle flexion angle within each CCL characteristic group.

Abbreviations: C, center; CCL, cranial cruciate ligament; Cd, caudal; Cr, cranial; max, maximum; min, minimum.

## 4 DISCUSSION

Identifying the rotation center of the osteotomized tibial fragment is critical for the rationale and accurate planning of TPLO.^3^, ^10^ This process requires precise intraoperative identification of the TIcE, which remains subjective and challenging.^10^, ^14^ This study indicates that positioning a hypodermic needle perpendicular to the sagittal plane at the MCL center, specifically at the interface of the articular surface with the most proximal medial edge of the tibial plateau while maintaining the stifle flexed at 135°, provides an accurate method for locating the mechanical center of the stifle in an experimental setting of acute complete CCL rupture. This approach aids in reliably identifying the TPLO fragment's center of rotation, thereby supporting our hypothesis and enhancing surgical accuracy while potentially reducing complication risks.

The MCL has long been used as the primary anatomical reference point to establish the center of rotation during TPLO, although evidence confirming its accuracy is limited.^14^, ^16^ Consequently, various adjuvant techniques have been adopted to optimize osteotomy positioning.^7^ In dogs, the MCL extends from the femur's medial epicondyle to the tibia's medial condyle and has an hourglass shape. Its course involves fusion with the stifle joint capsule and close association with the medial meniscus.^18^, ^22^ Given its positioning, the MCL theoretically aligns with the joint's rotational center, establishing it as a useful surgical reference.^13^, ^14^, ^15^, ^16^ The results of this study substantiate the MCL's utility as an indirect reference point for the osteotomy center during TPLO, with the noted variation based on the stifle's range of motion.

An error in identifying the rotation center can significantly impact the TPA and undermine the primary objective of plateau leveling,^10^ leading to a cascade of procedural complications affecting tibial tuberosity geometry, fragment rotation, and implant placement.^9^, ^10^, ^11^, ^23^ A key concern when osteotomy is not centered in TIcE is tibial long axis shift. This factor is a notable advantage of correctly performed TPLO over other tibial osteotomy techniques.^9^, ^24^ Using the cranial MCL edge as a reference may enlarge the final TPA and decrease the tibial tuberosity size, while caudal positioning can reduce bone stock for plate placement and undersize the final angle of the tibial plateau;^10^ however, clinical studies are needed to determine the true impact of these distances on TPLO outcomes and to define an acceptable margin of error in osteotomy centering. A recent study found that distal rotation center displacement increased quadriceps tensile strength, which could overload the patellar ligament, underscoring the need for precise osteotomy centering.^25^ Our study used the tibia's medial articular edge as a proximodistal reference point.

Our findings reveal that the range of joint motion and tibial craniocaudal instability relative to the femur significantly influence TIcE identification accuracy. The hypodermic needle marker, easily replicable intraoperatively, should be inserted perpendicular to the sagittal plane and positioned at the MCL center width, with the stifle flexed at 135° when the CCL is ruptured and at 90° when intact. In the results, the CCL‐MT‐Cr‐90° group also had a median of 0; however, its mean was significantly higher than that of the CCL‐MT‐C‐135° group, indicating greater accuracy in the latter. Clinically, however, the Cr‐90° positioning may still be useful, particularly in cases where excessive medial buttress in chronic conditions obstructs visualization of the medial collateral center. Conversely, in recent research, authors discouraged using the MCL center as a reference after finding that osteotomies shifted cranially from the original plan, with a 14.2% error rate observed in intact CCLs at a 90° stifle angle.^14^ Unlike our methodology, they measured the distance between the MCL‐referenced center and the planned osteotomy center (TIcE) in both the craniocaudal and proximodistal directions. Additionally, the error was expressed as a percentage relative to the planned TPLO blade size for each case.^14^

While MCL insertion points in dogs tend to be consistent, ligament tension and positioning vary based on stifle angulation.^18^, ^19^ The MCL is taut during extension but relaxes during flexion.^17^, ^26^ Additionally, a bursa at the tibial condyle facilitates MCL movement during extension and flexion.^18^ This flexibility explains the MCL's positional change relative to the tibia during extension. In both CCL conditions, the mean values of *d* were lower at 135° flexion than at 90°, suggesting a tendency for cranial displacement of the MCL during joint extension. Cranialization of the MCL was observed at 135° of flexion, in both CCL conditions, indicating a tendency toward cranial displacement with joint extension. This tendency resulted in lower mean values of “*d*” at 135° flexion compared with 90° means. Additionally, the higher mean values in the CCL‐MT groups compared with the CCL‐In groups may result from tibial cranial translation, as the MCL does not fully accommodate bone displacement.^27^ Because TPLO is primarily used to treat cruciate disease, the transected group more closely resembles clinical scenarios, whereas the intact group can represent cases of partial CCL injury.^28^

Additional anatomical landmarks are often used to enhance osteotomy positioning accuracy during TPLO planning and execution.^16^, ^29^ References such as the cranioproximal tibial tuberosity (patellar ligament insertion) and the caudal edge of the proximal tibia are used to determine specific distances to guide saw blade placement at the TIcE center.^5^, ^12^, ^16^ However, even with these landmarks, the eccentricity distance between the planned and actual center can be observed. Previous studies have demonstrated a tendency toward caudodistal eccentricity when using predetermined distance methods, such as D1/D2, for TPLO.^5^, ^16^ Precision in this procedure can be influenced by several factors, including jig and cutting guide use, blade model, and surgeon skill level.^12^, ^16^, ^30^ Improving accuracy in identifying the true center of rotation for the osteotomized fragment, using a direct anatomical reference from the intercondylar eminence (TIcE), could substantially enhance procedural success. Combined with other techniques, this approach can serve as an intraoperative double‐check to ensure the correct TPLO cut positioning.

Despite its longstanding establishment over the past three decades,^3^ TPLO continues to be a subject of considerable study aimed at refining both preoperative planning and intraoperative execution. Research has focused on preoperative stages, such as TPA measurement accuracy,^31^ and intraoperative phases, including precision in osteotomy execution and improved anatomical referencing.^14^, ^29^, ^30^ When the center of rotation is misidentified during surgery, clinical outcomes can suffer significantly,^10^ raising the likelihood of complications, such as tibial tuberosity fractures and tibial long axis shift.^8^, ^9^, ^24^ Therefore, the importance of accurately identifying the center of rotation during TPLO is once again highlighted. Although this study has identified the optimal reference point for locating the TIcE, a standard deviation of approximately 2 mm suggests individual variation that may affect clinical results.

These findings should be interpreted considering some limitations. First, the study was conducted exclusively on dogs within a specific size range, limiting the generalizability to smaller or larger breeds. Due to the low variation in TPA among study cases, an objective correlation between this variable and the results could not be determined. Another limitation was the inability to assess proximal‐distal needle variation due to difficulties in establishing a distance standard in cadaveric joints, where the articular space is substantially reduced. Additionally, the ex vivo models with intact CCL and iatrogenic ruptured may not fully represent a clinical scenario and do not include cases of partial rupture, as CCL disease patients, particularly chronic cases, may exhibit periarticular fibrosis that potentially affects MCL mobility. Although the intact CCL model is considered a limitation, it can serve as an anatomical reference for TiCE in future tibial surgeries for other conditions. Future studies should aim to validate these findings in clinical settings.

In conclusion, positioning a needle perpendicular to the sagittal plane at the MCL's center width, with the stifle flexed to 135°, provides a precise method for identifying the osteotomy center in TPLO procedures in dogs. This approach may improve procedural accuracy, broaden intraoperative verification parameters, and reduce complication risks associated with TPLO.

## AUTHOR CONTRIBUTIONS

Alcântara BM, DVM, PhD: Designed the study, conducted the study and radiographic measurements, compiled and interpreted data, and drafted and revised the manuscript. Minto BW, DVM, PhD: Supervised and codesigned the study, interpreted data, and assisted with manuscript writing. Carrera ALC, DVM, MS: Participated in study design, conducted the study and radiographic measurements, and revised the manuscript. Faustino RCS, DVM, MS: Contributed to study design, conducted the study and radiographic measurements, and revised the manuscript. Lopes LCM, DVM: Assisted in study design, conducted the study, interpreted data, and revised the manuscript. Dias LGGG, DVM, PhD: Contributed to study design, performed data analysis, and revised the manuscript. All authors critically reviewed the manuscript, endorsed the final version, and confirmed their contributions. Each author is fully aware of their respective contributions and is confident in the integrity of all parts of the work.

## CONFLICT OF INTEREST STATEMENT

The authors declare no conflict of interest.
